# Metal species involved in long distance metal transport in plants

**DOI:** 10.3389/fpls.2014.00105

**Published:** 2014-03-25

**Authors:** Ana Álvarez-Fernández, Pablo Díaz-Benito, Anunciación Abadía, Ana-Flor López-Millán, Javier Abadía

**Affiliations:** Plant Nutrition Department, Aula Dei Experimental Station (CSIC)Zaragoza, Spain

**Keywords:** metals, metal complexes, phloem, transport, xylem

## Abstract

The mechanisms plants use to transport metals from roots to shoots are not completely understood. It has long been proposed that organic molecules participate in metal translocation within the plant. However, until recently the identity of the complexes involved in the long-distance transport of metals could only be inferred by using indirect methods, such as analyzing separately the concentrations of metals and putative ligands and then using *in silico* chemical speciation software to predict metal species. Molecular biology approaches also have provided a breadth of information about putative metal ligands and metal complexes occurring in plant fluids. The new advances in analytical techniques based on mass spectrometry and the increased use of synchrotron X-ray spectroscopy have allowed for the identification of some metal-ligand species in plant fluids such as the xylem and phloem saps. Also, some proteins present in plant fluids can bind metals and a few studies have explored this possibility. This study reviews the analytical challenges researchers have to face to understand long-distance metal transport in plants as well as the recent advances in the identification of the ligand and metal-ligand complexes in plant fluids.

## Introduction

To reach their final destination within the plant (e.g., organelles such as chloroplast or mitochondria) micronutrients taken up from the growth medium, including metals such as Fe, Mn, Zn, and Cu, must follow a complex path through a number of different plant compartments and membrane systems (Clemens et al., 2002; Colangelo and Guerinot, 2006; Briat et al., 2007; Haydon and Cobbett, 2007; Curie et al., 2009; Puig and Peñarrubia, 2009; Conte and Walker, 2011; Sinclair and Krämer, 2012). The vascular system, including the xylem and phloem conduits, is an essential segment for long distance translocation of micronutrients within this path. It has long been proposed that a significant fraction of metals would be present in plant fluids not as free ions but in less reactive chemical forms, e.g., non-covalently bound to organic compounds, to prevent uncontrolled binding and also because free metals often exert some degree of toxicity. The formation of metal complexes provides both solubility and shielding during long-distance transport, since the metallic atom is enveloped by an array of bound molecules or anions (the so-called ligands; in this review only ligands consisting in organic molecules are considered), which donate one or more electron pairs to the metal to form the complexes.

Indirect evidence for long distance, organic ligand-assisted transport of metals has been extensively reported. Possible ligand candidates are a range of small molecules, including organic acids -carboxylates- such as citrate (Cit) and malate (Mal), amino acids [including nicotianamine (NA), histidine (His), cysteine (Cys) and high-affinity Fe(III) chelating compounds derived from NA called phytosiderophores (PSs), such as mugineic (MA) and 2′-deoxymugineic (DMA) acids], as well as peptides and proteins (e.g., metallothioneins). In the case of toxic metals such as Cd, Hg, and others, plants also respond by synthesizing compounds such as the poly-glutathione peptides phytochelatins (PCs) that decrease the amount of free metal ions in plant fluids. Recent papers have reviewed the roles of NA (Curie et al., 2009; Clemens et al., 2013) and PCs (Pal and Rai, 2010) in plant metal homeostasis, the intra- and extracellular excretion of carboxylates (Meyer et al., 2010) and the plant metallo-chaperones (Tehseen et al., 2010). During the last decade, the identification of many genes involved in plant long-distance metal transport has also been achieved, and many reviews have covered this issue in relation to either several metals (Colangelo and Guerinot, 2006; Haydon and Cobbett, 2007; Krämer et al., 2007; Curie et al., 2009; Palmer and Guerinot, 2009; Puig and Peñarrubia, 2009; Krämer, 2010; Waters and Sankaran, 2011) or to specific ones such as Fe (Briat et al., 2007; Kim and Guerinot, 2007; Morrissey and Guerinot, 2009; Conte and Walker, 2011; Sperotto et al., 2012; Thomine and Vert, 2013), Zn (Sinclair and Krämer, 2012), Cu (Yruela, 2009), Mn (Pittman, 2005), and Cd (Mendoza-Cózatl et al., 2011).

To date, analytical data on the actual metal complexes existing in plant fluids are still scarce. This is relevant, because the chemical forms in which a metal occurs in solution (metal speciation) affect solubility, precipitation, acid/base equilibria, electron-transfer reactions, diffusivity, ability to undergo photolysis and others. The metal species existing in any given compartment determine biological activity, including the capability to be a substrate for membrane transport proteins involved in loading and unloading to xylem and phloem, as well as the possibility that metal toxicity can occur. This review summarizes the current knowledge on metal species occurring in plant fluids [xylem sap, phloem sap and other fluids such as apoplastic fluid and embryo sac liquid (ESL)], and discusses general problems relevant to these studies as well as the methodological approaches currently used.

## Methods for plant fluid sampling and inherent problems

Metal translocation within plants is dynamic, with the composition of plant fluids often changing with time. This mandates to establish an adequate standard sampling protocol, including sampling time and others, which must be applied to all samples so that results are fully comparable. A major limitation in the analysis of plant fluids is the need for samples of adequate purity and in sufficient volume to carry out measurements. The purity of the fluid samples must always be assessed by measuring cytosolic and/or vacuolar components associated to cell rupture. The activities of cytosolic marker enzymes, such as Mal dehydrogenase (*mdh*) or others (López-Millán et al., 2000), or the concentrations of K as a vacuolar marker (Lohaus et al., 2001; Barabasz et al., 2012) are generally used with that purpose.

Xylem sap, the fluid contained in xylem vessels -composed of tracheary elements separated by rather large perforation plates-, is relatively easy to sample (reviewed by Alexou and Peuke, 2013). The most frequently used technique is de-topping plants and letting xylem to bleed after discarding the first drops (López-Millán et al., 2012). Other techniques involve centrifugation of excised stems (López-Millán et al., 2000) or the use of a Schölander pressure chamber (Larbi et al., 2003). With any of these extraction techniques the xylem sap volume sampled could reach several hundreds of μL.

The phloem sap, the fluid contained in sieve cells—separated by sieve plates—, is a special case, because there is still controversy about changes in composition induced by wounding (Atkins et al., 2011) and the presence of different types of phloem saps (Zhang et al., 2012). The purity of the phloem sap is usually assessed by measuring sugar concentrations and/or evaluating the presence of Rubisco proteins or transcripts (Giavalisco et al., 2006; Rodríguez-Medina et al., 2011; Lattanzio et al., 2013). Other authors consider pH values around 8.0 indicative of phloem purity (Ando et al., 2013). Some methods in use for phloem sap sampling (reviewed by Dinant and Kehr, 2013) are those involving an incision near the inflorescence (in *Cucurbitaceae*, *Brassicaceae* and some *Lupinus* species; Lattanzio et al., 2013), those using aphid or leaf-hopper stylectomy (with the disadvantage of resulting in minute phloem sap volumes, ≤1 μL per cut stylet; Ando et al., 2013), or by exudation of excised shoots maintained at >80% relative humidity in a closed chamber (the latter being only a qualitative approach; Marentes and Grusak, 1998). When using insect stylectomy, evaporation during sampling is a relevant issue; accurate determination of the phloem volume has been recently achieved by measuring the droplet diameter as it forms on the tip of the severed aphid stylet (Palmer et al., 2013).

The fluid in the apoplastic space in the leaf (free space outside the plasma membrane) is an interface between the xylem and the symplast. Some methods for leaf apoplastic fluid sampling involve direct centrifugation of leaves without petiole (previously centrifuged at low speed to remove xylem sap from the mid vein; López-Millán et al., 2000) or by using a Schölander pressure chamber (Larbi et al., 2003). Other authors infiltrate the leaves with a solution under vacuum and then obtain by centrifugation a leaf apoplastic wash fluid (Lohaus et al., 2001). Since the latter procedure leads to a dilution of the apoplastic fluid, concentrations should be corrected considering estimations of apoplastic volumes occupied by water and air, using vacuum infiltration with a ^14^C-sorbitol labeled solution and silicone oil, respectively (Lohaus et al., 2001).

Recently, the ESL has been successfully used to study the processes of metal transport in pea seeds (Grillet et al., 2014). This liquid is enriched by the bulk flow of nutrients delivered by the seed coat and feeds the embryo.

## Methodological approaches to unravel metal species occurring in plant fluids

Until recently, researchers had to rely on indirect methods to putatively identify the chemical forms of metals occurring in plant fluids. These methods were based on separate measurements of the concentrations of metals and possible ligands and the prediction of the existent chemical species by means of titration in “artificial” saps (Liao et al., 2000; Irtelli et al., 2009; Alves et al., 2011) or *in silico* by using a set of known, experimentally determined stability constants of metal-containing complexes and chemical speciation software; this was always done assuming that chemical equilibrium was achieved (White et al., 1981; López-Millán et al., 2000; Callahan et al., 2006; Harris et al., 2012). However, chemical equilibrium is unexpected in plant fluids, since continuous changes in composition usually occur in these dynamic plant compartments.

Molecular biology approaches have provided key information on putative metal ligands and metal complexes that could play a crucial role in inter- and intra-organ metal transport (see reviews by Briat et al., 2007; Haydon and Cobbett, 2007; Kim and Guerinot, 2007; Milner and Kochian, 2008; Palmer and Guerinot, 2009; Pal and Rai, 2010; Waters and Sankaran, 2011; Leitenmaier and Küpper, 2013; Thomine and Vert, 2013). Genotypes with loss-of-function of genes involved in the trafficking of metals, ligands and/or their metal complexes, as well as metal hyper-accumulator (e.g., *T. caerulescens*) plant species have been studied. Other approaches include mutant and transgenic lines with impaired or enhanced metal ligand synthesis; a classical example of impaired synthesis is the tomato mutant “*chloronerva*,” which lacks NA (Pich and Scholz, 1996). Transgenic genotypes have also been constructed where the synthesis of ligands such as PCs is restricted to a specific tissue (Gong et al., 2003).

Another approach is searching for molecules with affinity for metals in plant fluids, using their immobilization by metal affinity chromatography (IMAC) followed by identification of the isolated molecules using some of the mass spectrometry (MS) techniques described below (Lattanzio et al., 2013). In this approach, the occurrence of the corresponding metal-ligand complex in plant fluids is only inferred from the presence of suitable ligands.

A further approach to identify metal complexes in plant tissues -including fluids- relies on the identification of the chemical environment surrounding the metal by means of synchrotron X-ray absorption spectroscopy (XAS) techniques such as X-ray absorption near edge spectroscopy (XANES) and extended X-ray absorption fine structure spectroscopy (EXAFS) (Lombi et al., 2011; Donner et al., 2012). This has been used for different metals such as Fe (Yoshimura et al., 2000; Terzano et al., 2013), Mn (Yun et al., 1998), Cu (Küpper et al., 2009; Song et al., 2013), Zn (Salt et al., 1999; Küpper et al., 2004; Trampczynska et al., 2010; Lu et al., 2013), Ni (McNear et al., 2010), Cd (Salt et al., 1995; Vogel-Mikus et al., 2010; Huguet et al., 2012; Yamaguchi et al., 2012), Hg (Carrasco-Gil et al., 2011, 2013; McNear et al., 2012), and Pb (Tian et al., 2010; Chu et al., 2012). This technique allows direct *in situ* metal speciation in plants, without any preliminary extraction or preparation (see reviews by Lombi et al., 2011; Donner et al., 2012; Sarret et al., 2013). However, spectra are difficult to interpret when more than two or three species are dominant, and these techniques are generally used to confirm the presence of known species rather than to find new ones (Monicou et al., 2009). Furthermore, the applications of these techniques have been usually restricted to tissues from metal hyper-accumulators, due to the relatively low sensitivity (metal concentration should be higher than 10 μg g^−1^ dry weight). In spite of that, some data on metal speciation have been reported for sap and vasculature tissues, although mainly in metal hyper-accumulator species. This has been done for Zn in *T. caerulescens* (Salt et al., 1999; Küpper et al., 2004; Trampczynska et al., 2010) and *Sedum alfredii* (Lu et al., 2013), for Cd in *Brassica juncea* (Salt et al., 1995) and *Arabidopsis halleri* (Huguet et al., 2012), and for Ni in *Alyssum murale* (McNear et al., 2010). Recently, the use of highly brilliant synchrotrons as X-ray sources has increased one order of magnitude the sensitivity of EXAFS for trace elements (e.g., below 1 μg g^−1^ dry weight for Fe), thus allowing speciation of Fe in tomato xylem sap (Terzano et al., 2013) and Cd around the vascular bundles in node I -the one beneath the panicle- in *Oryza sativa* (Yamaguchi et al., 2012). Unfortunately, artifacts can arise from high intensity radiation damage to the sample (i.e., degradation of Fe(III) complexes with carboxylates and NA, as reported in Terzano et al., 2013).

Recent advances in analytical techniques, and specifically in MS, have enabled a new insight on metal speciation. The use of highly selective and sensitive molecular and metal-specific MS techniques has allowed the identification and quantification of individual, well-defined metal species in plant tissues (including metabolites and proteins), even at sub-nM levels (Meija et al., 2006; Monicou et al., 2009). These approaches generally use hyphenated techniques based on the separation of compounds by high-resolution techniques [e.g., liquid chromatography (HPLC) and capillary electrophoresis (CE)] and the determination of the metals and/or metal complexes by MS techniques [including inductively coupled plasma MS (ICP-MS), electrospray MS (ESI-MS), and others]. These new methodologies (reviewed by Monicou et al., 2009; Khouzam et al., 2012) offer unique advantages for the *de novo* identification of metallo-metabolites occurring at low concentrations, in particular in plant materials such as plant fluids that do not require multi-step extraction from the plant tissues. Metabolite structures can be elucidated using the empirical formulas of the parent compound and fragment ions (data provided by high-resolution and high-accuracy MS) and the lineage of fragment ions observed in tandem MS or multistage MS (MSn). Furthermore, complexes with metals having more than one stable isotope, such as Fe, Zn, Cu, Cd, and Hg, provide metal-specific isotopic signatures that allow for identification of MS signals corresponding to metal containing molecules (see an example for metal-NA complexes in Rellán-Álvarez et al., 2008). In the five past years, methods based on these analytical techniques have achieved the direct identification of several metal complexes with possible relevance in long distance transport, including carboxylate complexes with Fe(III) and Ni(II), NA and PS complexes with Fe(III), Fe(II), Ni(II), Co(II), Mn(II), Cu(II) and Zn(II), and His complexes with Cu(II) (Weber et al., 2006; Xuan et al., 2006, 2007; Rellán-Álvarez et al., 2008, 2010; Dell'mour et al., 2010; Köster et al., 2011a,b; Tsednee et al., 2012). However, until now only a few of these metal-ligand complexes have been really found in plant fluids (see below).

Another MS technique, high-precision multi-collection-ICP-MS (developed 20 years ago), which yields high-precision measurements of stable isotopes of transition elements (e.g., Fe, Cu, and Zn), has allowed the study of their fractionation during plant uptake and translocation (reviewed by von Blanckenburg et al., 2009). This technique provides information on the mechanisms involved in metal partitioning within the plant, but not on metal speciation or specific binding environments. Recent studies on fractionation of Fe and Cu stable isotopes, using plants relying on Strategy I or II for Fe uptake, have revealed that both metals undergo redox cycling during root-to-shoot translocation in Fe-deficient Strategy I plant species, but not in Strategy II ones (Guelke-Stelling and von Blanckenburg, 2007, 2012; Kiczka et al., 2010; Ryan et al., 2013). A recent *in silico* study predicted that redox Fe changes affect Fe isotopic fractionation, with Δ^56^Fe (^56^Fe/^54^Fe) being 3‰ heavier in Fe(III)-PS than in Fe(II)-NA (Moynier et al., 2013). Even in the absence of redox Fe changes, changes in speciation alone would create up to 1.5‰ differences in Δ^56^Fe: Fe(III)-PS is up to 1.5‰ heavier than Fe(III)-Cit and Fe(II)-NA is up to 1‰ heavier than Fe(II)-Cit (Moynier et al., 2013). These estimations are in agreement with the fact that roots of Strategy-II plants, which rely on Fe(III)-PS uptake, are isotopically heavier (by about 1‰) than the shoots, where Fe had presumably been transported as Fe(III)-Cit in the xylem or Fe(II)-NA in the phloem. Isotopic variations observed between younger and older leaves could also be explained by the occurrence of Fe acquisition *via* xylem and phloem (Moynier et al., 2013). Zinc sequestration in roots is mediated by a number of mass dependent processes that favor the heavier isotopes, including binding to cell walls, precipitation in intercellular spaces, binding to high affinity ligands in the root cell and sequestration in the vacuole (Aucour et al., 2011; Caldelas et al., 2011). Translocation processes of Zn within the plant also lead to significant fractionation of Zn stable isotopes, since shoots of several plant species were enriched in light isotopes, with the tallest species showing the largest fractionation in the youngest leaves (Moynier et al., 2009). This effect can be modified by the Zn status in those Zn hyper-accumulators that accumulate Zn mainly in the roots (e.g., *A. halleri*), due to the large fractionation that occurs before shoot Zn's re-translocation.

## Metals in plant fluids

The study of long distance metal transport has traditionally used differences in metal accumulation ratios (root to shoot metal content ratios) within the plant, considering different scenarios (i.e., growth conditions, genotypes, etc.). Only recently, high-throughput elemental analysis technologies have allowed the characterization of the full ionome in a large number of lines of several plant species (www.ionomicshub.org) (Baxter et al., 2008; Singh et al., 2013). This information, in combination with bioinformatics and genetic tools, has yielded candidate genes coding for transporters as well as gene networks involved in long distance metal transport.

Direct analyses of metals in plant fluids have been carried out in a number of studies, and their concentrations are generally in the μM range (Table 1). Therefore, the use of sensitive analytical techniques such as graphite furnace atomic absorption spectroscopy (GAAS) or inductively coupled plasma-mass spectrometry (ICP-MS) is mandatory for their determination. Prior to the analyses and immediately after the sampling of plant fluids, metals are usually stabilized in the solution by acidifying it. To avoid metal cross-contamination, high purity acids (ultra trace analysis quality grade) should be used for sample acidification, and also for sample digestion and cleaning of all materials (Husted et al., 2011; Ando et al., 2013). Concentrations found in the xylem sap, phloem sap and leaf apoplastic fluid of different plant species are in the ranges 4–168 μM for Fe, 0.5–245 μM for Zn, 0.3–30 μM for Cu, 4–400 μM for Mn and nd–0.1 μM for Cd and Ni (Table 1). These concentrations usually increase when either the metals are in excess in the plant growth medium or when plants (specially those genotypes known as metal stress tolerant) are treated with control metal concentrations after a period of metal deficiency. In these cases concentrations may reach 120–177 μM Fe in tomato xylem sap (Orera et al., 2010; Rellán-Álvarez et al., 2010, 2011a), 148 μM Zn in sugar beet xylem sap (Sagardoy, 2012), 43 μM Cu in *O. sativa* phloem sap (Ando et al., 2013), 2300 μM Mn in *O. sativa* leaf apoplastic fluid (Führs et al., 2010) and 1–100 μM Cd in *O. sativa*, *B. vulgaris*, and *S. lycopersicum* (Mori et al., 2009; Kato et al., 2010; Sagardoy, 2012). Higher concentrations of metals in plant fluids are also found in hyper-accumulator species: up to 450 μM Ni (Mari et al., 2006) and 524 μM Zn (Lasat et al., 1998) in *T. caerulense*, up to 1750 μM Ni in *Alyssum lesbiacum* (Kerkeb and Krämer, 2003) and up to 100 μM Cd and 486 μM Zn in *A. halleri* (Ueno et al., 2008) (Table 1). Altered levels of metals in plant fluids are also found in mutant genotypes as well as in transgenic plants overexpressing or losing the function of either one or several gene components of the uptake, sequestration or transport mechanisms (Nakamura et al., 2008; Ishimaru et al., 2011; Sasaki et al., 2011). The addition of protein synthesis inhibitors or ligands to the growth media has also a large effect on the metal concentrations in plant fluids. For instance, the addition of cycloheximide to the hyper-accumulator *A. lesbiacum* led to a 70% reduction of the xylem sap Ni concentration, whereas the addition of His to the non-accumulator *B. juncea* increased Ni in xylem sap (Kerkeb and Krämer, 2003).

**Table 1:** **Metal concentrations (in μM) in xylem sap, leaf apoplastic fluid and phloem sap in different plant species**.

**Species**	**Stress**	**Fe**	**Mn**	**Cu**	**Zn**	**Ni**	**Cd**	**References**
**XYLEM SAP**								
*A. lesbiacum*^H^	Ni					nd–1750^S^		Kerkeb and Krämer, 2003
*A. serpyllifolium*^H^	Ni					1000^S^		Alves et al., 2011
*A. halleri*^H^	Cd				486^S^		nd–100^S^	Ueno et al., 2008
*A. thaliana*		5^G^–10						Durrett et al., 2007
*B. vulgaris*	Fe	2^S^–6						López-Millán et al., 2000
	Fe	2^S^–4–11^S^^A^						Larbi et al., 2010
	Fe	4^S^–15–30^S^^A^						Orera et al., 2010
	Zn	5–9^S^			20–148^S^			Sagardoy, 2012
	Cd	5–11^S^			20–26^S^		<0.1–10^S^	Sagardoy, 2012
*B. carinata*	Cu			0.3–1^S^				Irtelli et al., 2009
*B. juncea*^H^	Ni					175^S^–500^S^^A^		Kerkeb and Krämer, 2003
	Cd						nd–50^S^	Salt et al., 1995
	Cd						nd–61^S^	Wei et al., 2007a
*B. napus*	Cd	10–15^S^	23–26^S^	0.6–4^S^	0.5–0.6^S^		nd–12^S^	Nakamura et al., 2008
	Cd	0.7^S^–7					nd–11^S^	Mendoza-Cózatl et al., 2008
*B. oleracea*			11–80		9–70			Shelp, 1987
*H. vulgare*	Fe	2^S^–30	8–58^S^	2–5^S^	5–20^S^			Alam et al., 2001
	Fe	nd–100^S^^A^						Kawai et al., 2001
*G. leucopteris*		20	4	0.6	14			Hocking, 1983
*G. max*	Zn	1^S^–6	7^S^–10	1^S^–6^S^	3–28^S^			White et al., 1981
*N. glauca*		11	4	2	22			Hocking, 1980
*O. sativa*		8^G^–14						Yokosho et al., 2009
	Fe	4^G,S^–6^S^						Yokosho et al., 2009
	Fe	2^S^–11						Kakei et al., 2009
	Cu	36^S^–40	107^S^–134–175^S^	5–9^S^	8–16^S^			Ando et al., 2013
	Cu			0.2^G^–1^G^–5				Deng et al., 2013
		30^G^–100	8–9^G^				nd	Ishimaru et al., 2011
	Cd	15^G^^S^–50^S^	8^G^^S^–8^S^				5^G^^S^–8^S^	Ishimaru et al., 2011
	Cd	40^S^–122^S^			12^S^–32^S^		0.1^S^–0.6^S^	Yoneyama et al., 2010
	Cd						3^S^–12^S^	Kato et al., 2010
	Cd						<0.1–2^S^	Uraguchi et al., 2009
*P. communis*	Fe	0.5^S^–4						Larbi et al., 2003
*P. persica*	Fe	1^S^–6						Larbi et al., 2003
*S. lycopersicum*	Fe	10^S^–150^S^^A^						Orera et al., 2010
	Fe	5^S^–20–121^S^^A^						Rellán-Álvarez et al., 2010
	Fe	5^S^–15–177^S^^A^						Rellán-Álvarez et al., 2011a
	Fe	4^S^–6^S^						Terzano et al., 2013
	Cu	13^G^^S^^A^–43	15–56^G^^S^	1^G^–6	2^G^^S^^A^–4^G^			Pich and Scholz, 1996
	Zn	3^S^–9	5^S^–7	1^S^–7	2–84^S^			White et al., 1981
	Zn	32–89^G^^S^			12–214^G^^S^			Barabasz et al., 2012
	Cd	2^S^–10			3–12^S^		nd–95^S^	Sagardoy, 2012
*T. arvense*	Zn				15–100^S^			Lasat et al., 1998
*T. caerulense*^H^	Zn				54–524^S^			Lasat et al., 1998
	Ni					nd–450^S^		Mari et al., 2006
**LEAF APOPLASTIC FLUID**								
*B. vulgaris*	Fe	3^S^–6						López-Millán et al., 2000
	Fe	7^S^–19–65^S^^A^						Larbi et al., 2010
*H. vulgare*	Mn		11–22^S^					Führs et al., 2010
*O. sativa*	Mn		400–2300^S^					Führs et al., 2010
	Mn		150^S^–600^G^^S^					Sasaki et al., 2011
*P. communis*	Fe	2^S^–5						López-Millán et al., 2001
*S. lycopersicum*	Zn	2^S^–4^G^^S^			1^S^–8^G^^S^			Barabasz et al., 2012
**PHLOEM SAP**								
*B. oleracea*			4–76		78–245			Shelp, 1987
*G. leucopteris*		83	24	8	66			Hocking, 1983
*N. glauca*		168	16	19	243			Hocking, 1980
*O. sativa*	Cu	54^S^–67–74^S^	7^S^–10	20^S^–30–43^S^	14^S^–22–24^S^			Ando et al., 2013
	Cd						nd–18^S^	Tanaka et al., 2007
	Cd	50^S^–63^S^			34^S^–115^S^		0.1^S^–0.5^S^	Yoneyama et al., 2010
	Cd						1^S^–3^S^	Kato et al., 2010
*R. communis*		40–64	8–12	16–28	40–74			Schmidke and Stephan, 1995
		37						Schmidke et al., 1999

Specific cases are marked as follows:

S High or low metal supply;

A High or low metal supply in combination with a chemical (e.g., histidine, ABA, metal resupply);

G Mutant or transgenic genotypes; and

H *Hyper-accumulator plant species. nd: not detected*.

Transport metal studies have also been approached using radioactive or stable isotopes. Radioactive metal isotopes supplied to plants can be detected by autoradiography, both at the whole plant and/or organ level (Cakmak et al., 2000a,b; Erenoglu et al., 2002). The application of ^59^Fe-citrate to source tissues of pea plants showed that Fe is complexed prior to phloem loading (Grusak, 1994). The high remobilization of ^109^Cd from the leaves and stem to the maturing grain was associated with high accumulation of Cd in durum wheat grain (Harris and Taylor, 2001). A more recent example is the use of ^109^Cd autoradiography in *A. halleri* (a Zn and Cd hyper-accumulator species) showing that after 3 weeks an enrichment of the leaf petiole, central vein and trichomes occurs, whereas after 9 weeks leaf edges were the most Cd-enriched tissues and Cd concentrations were lower in regions along leaf vascular bundles (Huguet et al., 2012). A visualization of metals at the microscopic level can be obtained using thin sections. For instance, ^109^Cd radioisotope imaging using 30-μ m thickness sections has demonstrated Cd xylem-phloem transfer immediately after root uptake in *O. sativa* (Kobayashi et al., 2013).

The use of stable metal isotopes and highly selective and sensitive ICP-MS detection has also allowed tracing long-distance transport of metals within plants. Metal sources labeled with stable isotopes and applied to the roots or to leaves were used to evaluate the translocation within the plant of ^67^Zn (Watmough et al., 1999; Benedicto et al., 2011), ^57^Fe (Rodríguez-Castrillón et al., 2008; Rojas et al., 2008; Orera et al., 2010), ^54^Fe (Orera et al., 2010), and ^207^Pb (Watmough et al., 1999). Recently, the application of stable isotopes combined with laser ablation ICP-MS has allowed to localize and quantify the metal tracer together with other metals in plant tissue thin sections. This has been applied for the quantitative imaging of Cu and other essential elements (such as K, Mg, Mn, P, S, and B) in the leaves of the Cu-tolerant plant *Elsholtzia splendens* treated with ^65^Cu (Wu et al., 2009). Whereas these are useful approaches for analyzing the spatial distribution or temporal changes of metals within the plant, only a snapshot of the distribution of metal at a given moment can be obtained. In contrast, the Positron-Emitting Tracer Imaging System technique, PETIS, allows for real-time, image quantification studies of the movement of elements in intact plants. The uptake and translocation of metals has been investigated in several graminaceous species using PET tracers ^52^Fe, ^52^Mn, ^62^Zn, and ^107^Cd (Tsukamoto et al., 2006, 2009; Suzuki et al., 2008; Fujimaki et al., 2010; Ishikawa et al., 2011). For instance, this tool demonstrated the direct translocation of Fe from roots to young leaves *via* phloem in *H. vulgare* (Tsukamoto et al., 2009).

## Ligands in plant fluids

Organic ligands involved in xylem and phloem metal translocation have been revealed mainly by physiological, genetic, and molecular studies, with the exception of some recent studies performing metabolite profiling using a combination of powerful analytical techniques (e.g., the LC-MS, GC-MS, and NMR analyses of latex of the Ni hyper-accumulator tree *Sebertia acuminata* by Callahan et al., 2008). Metal transport is associated with the occurrence in plant fluids of different classes of metal ligands, including: (i) compounds with just only O atoms as electron donors, such as different carboxylates (mainly Cit and Mal but also some less known ones such as methylated aldaric acid Callahan et al., 2008) and some *ortho*-dihydroxy phenolic compounds (e.g., protocatechuic acid; Ishimaru et al., 2011); (ii) compounds with O and N atoms as electron donors, such as amino acids (proteinogenic ones such as His and non-proteinogenic ones such as NA) and PSs (e.g., MA and DMA); and (iii) compounds with S atoms in which at least one of them acts as an electron donor, such as Cys, S-containing peptides (e.g., glutathione and their derivatives, PCs) and Cys-containing proteins (e.g., metallothioneins).

Carboxylates are usually found in the μM-mM range in plant fluids, whereas NA, DMA, His, PCs and others are generally in the μM range (examples are given in Table 2). Difficulties in determining ligands in plant fluids are inherent to their ability to form metal complexes. The total concentration of ligand (often very low) is distributed in different chemical forms: uncomplexed, complexed to different metals and even complexed to specific metals in different stoichiometries. Therefore, to prevent errors in ligand quantification, the conditions used during sample extraction, treatment and analytical determination seek either the complete dissociation of the existing metal-complexes (while preventing the formation of new ones) or the quantitative formation of the metal complexes [e.g., Fe(III)-PSs]. Also, derivatization of the ligands may be used. An example of the latter is the use of compounds such as fluorenylmethyloxycarbonyl to protect the amino groups of NA by blocking ligand atoms involved in metal complexation (these compounds also tag the ligand with a moiety that favors its detection) (Wada et al., 2007).

**Table 2:** **Metal ligand concentrations (malate and citrate in mM and the rest in μM) in xylem sap, leaf apoplastic fluid and phloem sap**.

**Species**	**Stress**	**Malate**	**Citrate**	**NA**	**DMA**	**His**	**PCs**	**References**
**XYLEM SAP**								
*A. lesbiacum*^H^	Ni					40–500^S^		Kerkeb and Krämer, 2003
*A. serpyllifolium*^H^	Ni	2^S^	0.5^S^			nd		Alves et al., 2011
*A. deliciosa*	Fe	0.2–0.4^S^						Rombolà et al., 2002
*A. halleri*^H^	Cd	0.02^S^	0.3^S^			12^S^		Ueno et al., 2008
*A. thaliana*			0.06^G^–0.09					Durrett et al., 2007
			0.2–0.9^G^					Schuler et al., 2012
*B. vulgaris*	Fe	2–30^S^	0.2–5^S^			nd–4^S^		López-Millán et al., 2000
	Fe	2–14^S^	1–3^S^					Larbi et al., 2010
	Zn	0.5^C^–2^S^	0.06–0.3^S^					Sagardoy et al., 2011
*B. carinata*	Cu			64–271^S^		17–140^S^		Irtelli et al., 2009
*B. juncea*^H^	Ni					40^S^–50		Kerkeb and Krämer, 2003
	Cd	0.02–0.23^S^	0.01^S^–0.7					Wei et al., 2007a
*B. napus*	Cd	0.7–0.9^S^	0.5–0.6^S^			66^S^–76		Nakamura et al., 2008
*H. vulgare*	Fe	0.01–0.03^S^	<0.01–0.02^S^		30–450^S^			Alam et al., 2001
	Fe				90^S^			Kawai et al., 2001
	Fe	0.8–5^S^	0.3–1.3^S^					López-Millán et al., 2012
*G. max*	Zn	0.04^S^–0.9	0.08^S^–1.5^S^			26^S^–62^S^		White et al., 1981
*N. glauca*						10		Hocking, 1980
*O. sativa*		0.15–0.18^G^	0.05^G^–0.1					Yokosho et al., 2009
	Fe	0.3^S^–0.4^G^^S^	0.1^G^^S^–0.2^S^					Yokosho et al., 2009
	Fe			10–18^S^	10–48^S^			Kakei et al., 2009
	Cu		0.08	28	34			Ando et al., 2013
*P. communis*	Fe	0.1–3	0.03–0.5^S^					Larbi et al., 2003
*P. persica*	Fe	0.7–2^S^	0.05–0.8^S^					Larbi et al., 2003
*Q. suber*	Cd	1–2^S^	0.5–1.2^S^					Gogorcena et al., 2011
*S. lycopersicum*	Fe	0.6–4^S^	0.04–0.6^S^					López-Millán et al., 2009
	Fe		0.01–0.17^S^					Rellán-Álvarez et al., 2010
	Cu			nd^G^^S^–20^S^				Pich and Scholz, 1996
	Zn	0.06^S^–0.8^S^	<0.04^S^–0.3^S^			6^S^–18		White et al., 1981
	Cd	0.1–0.3^S^	0.01^S^–0.03–0.06^S^					Sagardoy, 2012
*T. arvense*	Zn	0.1–0.3^S^	nd			110–140^S^		Lasat et al., 1998
	Ni					nd^S^–57		Persans et al., 1999
*T. caerulense*^H^	Zn	0.1–0.2^S^	nd		nd			Lasat et al., 1998
*T. goesingense*^H^	Ni					8–18^S^		Persans et al., 1999
**LEAF APOPLASTIC FLUID**								
*B. vulgaris*	Fe	0.7–2^S^	0.7–4^S^			<1–4^S^		López-Millán et al., 2000
	Fe		0.2–4^S^					Larbi et al., 2010
*P. communis*	Fe	1.6^S^–2	0.8–1.8^S^					López-Millán et al., 2001
**PHLOEM SAP**								
*C. maxima*		1.6	2.1					Fiehn, 2003
*L. texensis*		5.5	1.1					Lattanzio et al., 2013
*N. glauca*						370		Hocking, 1980
*O. sativa*	Cu		>0.08	66	152			Ando et al., 2013
	Cd		>0.001–>0.002^S^	66–83^S^	152–176^S^		nd–63^S^	Kato et al., 2010

S High or low metal supply;

G Mutant or transgenic genotypes; and

H *Hyper-accumulator plant species*.

*Nd, not detected; NA, nicotianamine; DMA, 2'-deoxymugineic acid; His, histidine; PCs, phytochelatins*.

Classical analytical methodologies for organic ligands were based on direct HPLC-UV/VIS analyses for carboxylates (López-Millán et al., 2000), NA (Pich et al., 1994), PSs (Alam et al., 2001) and also for PCs (in the latter case, HPLC-UV/VIS is used in combination with pre- or post-column derivatization with UV/VIS-active tags, such as Ellman's reagent). New methodologies, based generally on HPLC-ESI-MS, have been developed using high resolution MS such as time-of-flight (TOF) MS for carboxylates (Rellán-Álvarez et al., 2011b and references therein) and NA and PSs (Wada et al., 2007; Kakei et al., 2009; Schmidt et al., 2011), Fourier transform ion cyclotron resonance-MS for NA and PSs (Weber et al., 2006), and quadrupole-TOF MS for NA and PSs (Tsednee et al., 2012). Other techniques have also been used, including CE coupled to UV/VIS (Xuan et al., 2007) or MS detection (Dell'mour et al., 2010), and GC-MS (for NA and/or PSs; Wirth et al., 2009; Rellán-Álvarez et al., 2011a). Nicotianamine and PSs have been analyzed, either using previous derivatization (Wada et al., 2007; Kakei et al., 2009; Schmidt et al., 2011) or by direct analysis (Xuan et al., 2007; Tsednee et al., 2012). In the case of PCs, analytical methods are also based on HPLC-MS techniques using either molecular detection (HPLC-ESI-MS and HPLC-ESI-MS/MS) or elemental detection (ICP-MS), and are often combined with derivatization (reviewed by Wood and Feldmann, 2012). For instance, N-(2-ferrocene-ethyl)maleimide is an electroactive pre-column tag that yields thiol-PCs conjugates, which can be separated and quantified by ESI-MS or ICP-MS detection, with detection limits for S at the nM level.

Quantification of ligands in plant fluids has been always done using external calibration with or without internal standardization. The latter is required when using HPLC-ESI-MS-based technologies, because the degree of ionization of a given analyte in different matrices can vary significantly and signal suppression (or enhancement) commonly occurs. Ideally, an isotope-labeled compound should be used as internal standard (IS) per each analyte to achieve accurate quantification; however, since isotope labeled compounds have a limited commercial offer and are quite expensive, either one or two isotope-labeled compounds or structural analogues are generally used. For instance, ^13^C-labelled Mal and succinate have been used for quantification of carboxylates (Rellán-Álvarez et al., 2011b). For quantification of NA and PSs, different ISs, including structural NA analogs such as nicotyl-lysine (Wada et al., 2007) and nicotine (Tsednee et al., 2012), or ^15^N-labelled NA produced using a recombinant *Schizosaccharomyces pombe* strain expressing NAS (Schmidt et al., 2011) have been used.

Since many carboxylates have more than one carboxylate group and some also have a α-hydroxycarboxylate binding center (i.e., α-hydroxy acids such as Cit and Mal), they can act as mono- or poly-dentate (bidentate and so on) ligands, and form complexes with several metals (e.g., Fe, Mn, Cu, Ni, Zn, Pb, etc.). Carboxylates with shorter chains or closely packed carboxyl groups with adjacent alcohol groups (α-hydroxy acids) form stronger complexes. These characteristics, as well as the increases in carboxylate levels found in some metal-stressed plants (Table 2), have supported that carboxylates could be associated to long-distance metal transport. For instance, Fe-deficiency causes a well-known increase in carboxylate concentrations (mainly Cit) in xylem sap (Abadía et al., 2002; Rellán-Álvarez et al., 2010) and leaf apoplastic fluid (López-Millán et al., 2000, 2001; Larbi et al., 2010). This occurs not only in several Strategy I plant species (Table 2; Abadía et al., 2002 and references therein; Rellán-Álvarez et al., 2010 and references therein) but also in Strategy II plant species (Alam et al., 2001; Yokosho et al., 2009; López-Millán et al., 2012), likely as a result of an increased anaplerotic C fixation mediated by the root phospoenolpyruvate carboxylase (López-Millán et al., 2000; Andaluz et al., 2002; López-Millán et al., 2012). This increased carboxylate flux in xylem sap would supply C to the Fe-deficient foliage that is deprived of C skeletons (López-Millán et al., 2000, 2012) and could also increase Fe supply via formation of Cit complexes with extracellular Fe pools (see below). This is in line with the use of citric acid in industry for cleaning and prevention of the clogging of pipes with colloidal and particulate Fe. An increased activity of citrate synthase (CS) and/or an overexpression of CS genes has been reported in plants grown with low Fe supply (e.g., *Beta vulgaris* López-Millán et al., 2000, *Pyrus communis* López-Millán et al., 2001, *A. thaliana* Thimm et al., 2001, *Actinidia deliciosa* Rombolà et al., 2002, *S. lycopersicum* López-Millán et al., 2009, *Pisum sativum* Jelali et al., 2010, *Malus xiaojinensis* Han et al., 2012 and citrus Martínez-Cuenca et al., 2013) and more recently in *M. xiaojinensis* plants grown with excessive Fe supply (Han et al., 2012). In fact, overexpression of an apple CS gene increased tolerance to Fe stress (low and excessive Fe supply) in transgenic *A. thaliana* and *Nicotiana tabacum* plants (Han et al., 2012, 2013). Increases in xylem carboxylate concentrations have also been described with other metal stresses, including excess of metals in crop (e.g., Zn in sugar beet Sagardoy et al., 2011 and Cd in tomato Sagardoy, 2012), and forest species (Cd in *Quercus suber* Gogorcena et al., 2011), as well as in metal hyper-accumulators (e.g., the Zn hyper-accumulator *T. caerulescens* Lasat et al., 1998, the Cd hyper-accumulator genotype of *B. juncea* L. Wei et al., 2007a and the Ni hyper-accumulator tree *S. acuminata* Callahan et al., 2008). It has been hypothesized that the increases in xylem carboxylates may constitute a general mechanism to cope with situations causing reduced photosynthetic activity (Sagardoy et al., 2011).

Catechols such as caffeic and protocatechuic acids are phenolic compounds with two adjacent hydroxyl groups in the aromatic ring, which have very high affinity for Fe(III). These compounds are involved in long-distance transport of Fe in *O. sativa*, since the mutant *phenolics efflux transporter* (*pez1*) had reduced concentrations of Fe, protocatechuic and caffeic acids in the xylem, along with increased root apoplasmic Fe (Ishimaru et al., 2011).

Histidine (His) is one of the strongest metal-coordinating ligands among the proteinogenic amino acids, and has three metal binding sites: carboxylate, α-amino and imidazole groups. The coordination to metals through the latter group forms rigid bonds and strong complexes, especially with Ni and Cu. However, evidence for a role of His in long-distance metal transport in plants is mostly related to Ni in the xylem of hyper-accumulators of the genus *Allysum*. Histidine (in the μM-mM range) and Ni xylem sap concentrations are significantly and linearly correlated in several *Alyssum* Ni hyper-accumulators (such as *A. lesbiacum*) in response to increased metal concentrations in the growth media (Krämer et al., 1996). This increased xylem loading of Ni is associated with constitutively higher root concentrations of His. Moreover, exogenous applications of His to either the roots or shoots of the non-accumulator plant species *Alyssum montanum* and *Brassica juncea* greatly increases the root-to-shoot mobility of Ni (Kerkeb and Krämer, 2003). In *A. lesbiacum* shoot His concentrations only increased when plants were exposed to Ni, and the levels of transcripts of the enzymes of the His biosynthesis pathway were constitutively higher in *A. lesbiacum* than in the non-accumulator *A. montanum*, especially for the first enzyme in the biosynthetic pathway, ATP-phosphoribosyltransferase (ATP-PRT) (Ingle et al., 2005). Moreover, the overexpression of an ATP–PRT cDNA in *A. thaliana* resulted in increases in shoot His and Ni tolerance (Wycisk et al., 2004). However, it has been recently reported that His does not play a role in Ni translocation in the xylem sap of *Alyssum* ssp. under field conditions (Alves et al., 2011; Centofanti et al., 2013). The Ni-His complex could occur in xylem sap under N-sufficient conditions, whereas under N-limited conditions, such as those usually found in the field, Ni translocation would occur as a free ion or complexed with carboxylates (Alves et al., 2011).

Nicotianamine (NA) and related molecules such as PSs are multi-dentate aminoacid chelators having more than one α-aminocarboxylate binding centers, which confer high affinity not only for Fe but also for other metals such as Zn, Cu, Mn, Ni, and Cd. Nicotianamine has affinity for Fe(III) and Fe(II), whereas PSs have an α-hydroxycarboxylate binding center that confers selectivity for Fe(III). Nicotianamine is ubiquitous in higher plants and present in all tissues, and is involved in plant metal trafficking (Curie et al., 2009; Clemens et al., 2013). In the NA-free tomato mutant *chloronerva*, which displays a strong interveinal chlorosis in young leaves, the long-distance transport of Cu but not that of Fe is impaired, indicating the importance of NA in Cu trafficking (Pich and Scholz, 1996). Unlike NA, PSs are restricted to grasses and secreted to the rhizosphere, and they are responsible for Fe and Zn acquisition (Suzuki et al., 2006). Both NA and PSs form metal stable complexes at the pH values occurring in plant fluids (von Wirén et al., 1999, 2000; Rellán-Álvarez et al., 2008). Furthermore, hydroxylated PSs such as MA and epi-MA have a higher affinity for Fe(III) than the non-hydroxylated DMA at the pH values from 5 to 7 typical of xylem sap, and this represents a competitive advantage when moving through slightly acid environments (von Wirén et al., 2000).

In xylem sap of *O. sativa*, NA and DMA concentrations are in the ranges 10–20 μM and 10–50 μM, respectively (Kakei et al., 2009), whereas in phloem sap higher concentrations were found, in the range of 66–83 for NA and 152–176 μM for DMA (Kato et al., 2010). Metal stresses caused increases in xylem sap NA or PSs concentrations in several species, including MA and DMA in Fe-deficient *H. vulgare* (Alam et al., 2001; Kawai et al., 2001), DMA in Fe-deficient *O. sativa* (Kakei et al., 2009), and NA in Cu-deficient Brassica (Irtelli et al., 2009) and Fe-deficient *Prunus persica* (Rellán-Álvarez et al., 2011a). Nickel-induced NA root-accumulation occurred in *T. caerulescens*, a Cd/Zn/Ni hyper-accumulator, but not in *T. arvense*, and this suggests that NA could be involved in Ni translocation *via* xylem in *T. caerulescens*, resulting in a higher capacity to transport Ni to shoots (Mari et al., 2006).

Phytochelatins are oligomers of the tri-peptide glutathione (-GluCysGly) and act as metal (Cd, Hg, Zn, and others) chelators through the thiol (-SH) group of Cys. Phytochelatins form a family of structures with increasing repetitions of the -Glu-Cys dipeptide units, followed by a terminal Gly, (-Glu-Cys)n-Gly or (-EC)n-Gly, where *n* generally ranges from 2 to 5 but can be as high as 11. A number of structural variants have been identified in a wide variety of plant species, and different metals, including Cd, Pb, Zn, and Hg, have been found to induce PCs production (reviewed by Pal and Rai, 2010). The occurrence of long-distance transport (either from shoot-to-root or from root-to-shoot) of PCs and some intermediates of their biosynthesis (e.g., γ-glutamylcysteine) during heavy-metal detoxification was first shown with transgenic and grafted Arabidopsis plants where PCs synthesis was restricted to specific tissues (Gong et al., 2003; Chen et al., 2006; Li et al., 2006). However, the direct determination of PCs in plant fluids has only been achieved more recently: glutathione and PCs were found in the phloem of Cd-treated *B. napus* (by HPLC coupled to both fluorescence and ESI-MS; Mendoza-Cózatl et al., 2008) and *O. sativa* (by CE-MS; Kato et al., 2010), and As-treated *R. communis* (by HPLC-ESI-MS; Ye et al., 2010). Lower concentrations of PCs (or trace levels) were found in the xylem of *B. napus* (Mendoza-Cózatl et al., 2008) and *R. communis* (Ye et al., 2010), suggesting that phloem is the major vascular system for PC-facilitated long-distance metal transport.

Proteins can also be involved in metal transport in fluids. A significant fraction of metals has been associated with the high molecular weight fraction in the phloem sap of *R. communis* (Fe Krüger et al., 2002) and *O. sativa* (Cu Ando et al., 2013 and Cd Kato et al., 2010). Among metal-binding proteins, metallothioneins (MTs) are low molecular weight (5–10 kDa), Cys-rich proteins, which are able to bind a variety of metals by the formation of mercaptide bonds with the numerous Cys residues present in the proteins (see review by Freisinger, 2011). Metallothioneins are implicated in several processes related to metal homeostasis, detoxification, distribution, and redox regulation, in particular under normal (non-stressed) physiological conditions. Evidence supports its role as metal carriers, mainly in the phloem sap. An up-regulation of MTs in *H. vulgare* was found during senescence (when metal remobilization occurs from senescing leaves), heavy metal treatments and Cu deficiency (Heise et al., 2007). Metallothioneins have been reported to occur in the phloem of *Apium graveolens* (Vilaine et al., 2003), *R. communis* (Barnes et al., 2004), *O. sativa* (Aki et al., 2008) and *L. texensis* (Lattanzio et al., 2013), when grown under non-stressed conditions. The induction of MTs (MT1) expression in leaf veins (and to a lesser extent in mesophyll cells) in response to Cu stress in *A. thaliana* suggest that this MT could be important for scavenging Cu in leaf veins (García-Hernández et al., 1998). Also, in hyper-accumulator plants, MTs could help detoxify the excess Cu accumulated by the high expression of the Cd/Zn ATPase HMA4 (Leitenmaier and Küpper, 2011, 2013).

## Challenges analyzing metal complexes in plant fluids

Several challenges are faced when studying metal speciation in plant fluids, because of the changes in metal speciation that may occur at sampling and/or during storage, and especially during sample preparation, separation and determination (Husted et al., 2011). Challenges when attempting the analysis of the metal chemical form(s) existing in a plant fluid occur because: (i) dynamic metal–ligand systems such as those in plant fluids inevitably include labile or transient metal species; (ii) biochemical processes such as enzymatic activities may cause degradation of metal complexes, (iii) metal species occur in plant fluids at very low concentrations (in the μM range; see below; Table 1), (iv) the metal complex distribution strongly depends not only on pH, but also on the metal-to-ligand ratios (Weber et al., 2006; Xuan et al., 2006, 2007; Rellán-Álvarez et al., 2008). The latter is specially important in plant fluids, since unlike stable metal chelates with the hexadentate ligands NA and PSs, which always occur with 1:1 stoichiometry, many of the known metal ligands existing in xylem and phloem saps (e.g., amino acids and carboxylates) may act as bi- and tri-dentate ligands, resulting in numerous metal-ligand species with different stoichiometries and charge states (see examples for Fe-Cit complexes in Silva et al., 2009; Rellán-Álvarez et al., 2010). For instance, in a solution with a 1:2 Fe:Cit ratio and pH 4, up to thirteen different Fe-Cit species were detected in aqueous solution by ESI-MS, whereas only two species occurred in a solution prepared at 1:100 Fe:Cit ratio at the same pH (Silva et al., 2009). Also, even for stable metal species, ligand exchange reactions may occur (altering the actual composition of the sample) at any step previous to detection, due to the presence of competing ligands and/or redox mediators. Ligand exchanges have already been reported in the cases of Fe(III)-NA (with Cit; Rellán-Álvarez et al., 2008) and Fe(III)-DMA (with NA; Weber et al., 2006). Some of these challenges are especially critical in separation-based methods (e.g., HPLC, CE), because the separation of the free ligand does change the metal-to-ligand ratio, and also because the pH may change considerably when organic solvent modifiers are used (Rellán-Álvarez et al., 2008, 2010; Köster et al., 2011a).

Sampling and storage procedures (temperature, light, etc.), can be considered as key aspects to preserve the metal species occurring in samples during the whole analytical process (reviewed by Mesko et al., 2011). Temperature needs to be as low as possible to reduce metal species transformation. For this purpose, lyophilization and shock-freezing in liquid N are the most common procedures used to preserve metal species in fluids. The latter is considered the safest technique to prevent metal species changes because it can be performed immediately at the sampling site and also because sample is stored in an inert gas atmosphere. Light can cause changes in metal speciation because it can induce electron transfer reactions affecting the stability of the metal complexes and also the structural integrity of the ligands. For instance, photochemical reduction of Fe(III) complexes with ligands such as di- and tri-carboxylic acids is well known (Bennett et al., 1982), and are accompanied by oxidative decarboxylation of the ligand. This issue could limit the use of irradiation with high intensity synchrotron X-rays for metal speciation (Terzano et al., 2013).

Finally, accurate quantification of metal species, generally performed either on-line or off-line after separation, is currently carried out using metal (ICP-MS) or molecular (ESI-MS) detection, in combination with isotope dilution analysis (IDA) that requires the use of an isotopolog of the analyte. When ICP-MS is used, a stable isotope of the metal is added after the separation of the metal complexes. For instance, Rellán-Álvarez et al. (2010) used ^57^Fe post-column addition to quantify Fe-Cit species in tomato xylem sap. When molecular detection such as ESI-MS is used, the isotopolog should be either a metal complex with a stable isotope-labeled ligand, or alternatively a structural analogue of the ligand. As mentioned above, the limited supply of stable isotope labeled ligands is an additional constraint for metal speciation.

## Metal species in xylem sap

Most of the studies exploring the chemical forms of metal complexes in plant fluids have been conducted using xylem sap. Metals occurring in the xylem sap may be preferentially complexed by the more acidic carboxylic acids (existing at concentrations from 2 to 9 mM in the xylem) rather than the much more basic amino acids (existing at concentrations from 1 to 3 mM in the xylem) due to the relatively acidic pH of this fluid, which is generally in the pH range from 5 to 6.5 (Harris et al., 2012).

A Fe(III)-Cit complex [tri-Fe(III), tri-Cit complex (Fe_3_Cit_3_)] was found for the first time in the xylem sap of tomato, using an integrated HPLC-MS approach, consisting in hydrophilic interaction liquid chromatography (HILIC) coupled to both ICP-MS and ESI-MS(TOF), combined with the use of stable isotope (^54^Fe) labeling; the identification was based on exact molecular mass, isotopic signature, Fe determination and retention time (Rellán-Álvarez et al., 2010). Citrate had been considered for many years a likely candidate for Fe xylem transport, but the possible Fe-Cit species in the xylem sap were only predicted from the co-migration of Fe and Cit during paper electrophoresis of xylem sap (Tiffin, 1966) or from *in silico* calculations (von Wirén et al., 1999; López-Millán et al., 2000, 2001; Rellán-Álvarez et al., 2008). The Fe_3_Cit_3_complex was only found in xylem samples with Fe concentrations above 20 μM (the limit of detection for the complex), such as those in Fe-deficient plants after Fe-resupply. The complex could not be detected in Fe-deficient and control plants, which have lower xylem sap Fe concentrations. The existence of other Fe-Cit complexes is likely, and the complex Fe_2_Cit_2_ was also detected in Fe-Cit standards along with Fe_3_Cit_3_, with the allocation of Fe between the two complexes depending on the Fe:Cit ratio. Since in plant xylem sap a wide range of Fe to citrate ratios could exist, it is likely that both Fe(III)-Cit species could occur in different conditions (Rellán-Álvarez et al., 2010). Later, other Fe-Cit species were found along with Fe_3_Cit_3_ in *H. vulgare* leaf extracts using HILIC coupled to high-resolution Fourier transform ion cyclotron resonance (FT-ICR) MS (Köster et al., 2011a). More recently, the Fe speciation in tomato xylem sap was assessed for the first time using XANES on a highly brilliant synchrotron (PETRA III, beamline P06; Terzano et al., 2013). Although this study confirmed the occurrence of Fe(III)-Cit and also found Fe(III)-acetate complexes in xylem sap, the authors indicated that complexes found could be artifacts as a result of the high intensity radiation used. Studies with *FRD* mutants (i.e., *Atfrd3* and *Osfrdl1*), which lack a protein responsible for efflux of Cit in cells of the xylem vasculature, also support the role of Cit as a major ligand responsible for Fe complexation (Durrett et al., 2007; Yokosho et al., 2009). These mutants showed leaf chlorosis and low levels of Fe in xylem and leaves, as well as decreased Cit levels of in the xylem sap. Taken together, all these studies support that Fe-Cit is responsible for the translocation of an important fraction of Fe to the shoot, and that FRD mediated-Cit efflux is required to sustain normal rates of root–shoot Fe delivery. More recently, it was shown that FRD mediated-Cit efflux is also a major player in mobility of Fe in inter-cellular spaces lacking symplastic connections (Roschzttardtz et al., 2011).

The possible role of NA in long-distance Fe transport in the xylem is still being explored (Curie et al., 2009). However, strong evidence supports that this amino acid is not essential for xylem Fe transport: the NA-deficient tomato mutant *chloronerva* does accumulate Fe in old leaves (Pich et al., 1994) and the *A. thaliana* NA synthase (NAS) quadruple mutant (with low levels of NA) also accumulates Fe in leaves (Klatte et al., 2009). Until now, Fe-NA chelates have not been detected in xylem sap (Rellán-Álvarez et al., 2010), and *in silico* and/or *in vitro* speciation studies tend to exclude NA as a possible xylem Fe carrier at the slightly acidic pH values typical of xylem (von Wirén et al., 1999; Rellán-Álvarez et al., 2008). However, it has been recently suggested that NA may play a role in long distance transport of Fe when carboxylates are in short supply, as it occurs in *FRD* mutants (Schuler et al., 2012) or in plant species with less acidic xylem such in field-grown *Prunus persica* trees (where the xylem sap pH is in the range from 6.5 to 7.5 Larbi et al., 2003; Rellán-Álvarez et al., 2011a). The most accepted role of NA is in intra-organ Fe distribution, where this ligand could be crucial for xylem Fe unloading. Iron distribution within leaves is impeded in the tomato mutant *chloronerva* (Pich et al., 1994) that also showed a lower presence of Fe(II) ions in the veins when leaves were analyzed by XANES (Yoshimura et al., 2000). This suggests that the occurrence of Fe as Fe(II)-NA complex in leaf veins is crucial for the intra-organ Fe allocation (Yoshimura et al., 2000). No transporter responsible for moving Fe-complexes into the xylem sap has been conclusively identified so far, but it has been suggested that Fe-NA could be effluxed into the xylem by a yellow-stripe-like (YSL) transporter (Colangelo and Guerinot, 2006). Regarding PSs, only a minor peak assigned to Fe(III)-DMA was found in press sap from the roots of Fe-deficient wheat plants by HILIC-ESI-MS (Xuan et al., 2006).

The Zn species present in the xylem are still an open question (Clemens et al., 2013). Three studies tackling Zn speciation in xylem sap were carried out using EXAFS or XANES with the hyper-accumulators *T. caerulescens* (Salt et al., 1999; Monsant et al., 2011) and *S. alfredii* (Lu et al., 2013). In all cases, although the major fraction of Zn consisted in free hydrated Zn^2+^ ions, the remaining fraction was coordinated with carboxylates such as Cit and Mal. The occurrence of Zn-Cit in xylem sap was also predicted by *in silico* studies in non hyper-accumulator species (White et al., 1981; Mullins et al., 1986). However, other EXAFS study indicated that most of the Zn in petioles and stems of *T. caerulescens* is present as Zn-His (Küpper et al., 2004) and a recent study (including the re-evaluation of previous EXAFS spectra from this species) proposes His as a Zn ligand within cells and NA as Zn chelator involved in long distance transport (Trampczynska et al., 2010). In a recent room temperature XANES study with *T. caerulescens* intact plants, Zn-His and Zn-phytate complexes were found in roots, whereas Zn(II)-Mal and Zn(II)-Cit were the major species in shoots (Monsant et al., 2011). *In vitro* metal exchange experiments also support the existence of the complex Zn(II)-NA in the xylem sap (Rellán-Álvarez et al., 2008). It has also been speculated that Zn would be associated with S ligands in Cys, GSH or PCs in hyper-accumulators (Milner and Kochian, 2008). In the Zn hyper-accumulator *A. halleri*, suppression of NA synthase (NAS2) resulted in strongly reduced NA root accumulation, and a concomitant decrease in root-to-shoot translocation of Zn (Deinlein et al., 2012). This study found NA and thiols as the dominant Zn ligands in the low molecular weight fraction of root extracts by using size-exclusion chromatography (SEC)-ICP-MS combined with off-line ESI-MS ligand detection in the Zn-containing LC fractions. The overexpression of *A. thaliana* ZINC-INDUCED-FACILITATOR1 (ZIF1) altered the subcellular partitioning of NA, which was accumulated in roots, and led to a Zn accumulation in roots at the expense of shoots (Haydon et al., 2012). This indicates that the formation of Zn(II)-NA could be responsible for Zn hyper-accumulation. However, the complex Zn(II)-NA has never been found in the xylem sap yet. In grasses, a Zn(II)-DMA complex was detected in press sap from roots of Fe-deficient wheat plants using HILIC-ESI-MS (Xuan et al., 2006).

The Cu(II)-DMA complex has been recently been found in xylem sap of *O. sativa* (Ando et al., 2013). The Cu(II)-DMA complex was identified by SEC combined with both off-line Cu determination (using GFAAS) and off-line molecular detection of the complex by ESI-MS, based on exact molecular mass and isotopic signature. In this study, the Cu, NA, and DMA xylem sap concentrations were 5, 28, and 34 μM, respectively (the molar ratio DMA:Cu was *ca*. 7). The same complex was already found in press sap from roots of Fe-deficient wheat plants, both using HPLC-MS (Xuan et al., 2006) and CE and UV-VIS detection (Xuan et al., 2007). The presence of Cu(II)-DMA is not unexpected, since it has a quite high stability constant (18.7; Murakami et al., 1989). Nicotianamine and DMA are present in comparable concentrations in the xylem, but Cu(II)-NA has not been found in xylem sap so far, in spite of having a similar stability constant to that of Cu(II)-DMA (18.6; Callahan et al., 2006). A preferential Cu complexation by DMA vs. NA was already predicted by *in silico* speciation (von Wirén et al., 1999). In plant species other than grasses, the low leaf Cu concentration found in the NA-free tomato mutant *chloronerva* is in strong support that Cu(II)-NA is involved in xylem Cu transport (Pich and Scholz, 1996). The EXAFS spectra of *T. caerulescens* shoots also suggested the presence of Cu(II)-NA (Mijovilovich et al., 2009). Further support for the role of NA in xylem Cu transport was obtained when the NA aminotransferase gene from *H. vulgare* was introduced into the non-graminaceous plant *N. tabacum*. When compared to wild type, these transgenic plants showed decreased Cu concentration in young leaves and flowers (Takahashi et al., 2003), attributable to the depletion of endogenous NA. The occurrence of a Cu-His complex has been reported in the xylem sap from the Ni hyper-accumulator *A. lesbiacum* by EXAFS (Krämer et al., 1996) and in *H. vulgare* leaf aqueous extracts by HILIC-FT-ICR/MS (Köster et al., 2011a).

Long-distance Ni transport has been demonstrated in hyper-accumulators (*Allysum* and *Thlaspi* species) coordinated with several ligands, including different carboxylates (e.g., Cit, Mal, etc.) and amino acids (e.g., NA and His). An in depth study of Ni ligands using a combination of advanced MS and NMR techniques in the latex of the Ni hyper-accumulator tree *S. acuminata* revealed the presence of Ni in complexes with methylated aldaric acid and Cit (Callahan et al., 2008). In this latex, containing 26% dry matter of Ni, Ni(II) was forming complexes of 1:2 stoichiometry [Ni(II):carboxylate] with these two carboxylates as well as with Mal, aconitate, erythronate, galacturonate, tartarate, aconitate, and saccharate. A Ni(II)Cit_2_ complex (accounting for 99.4% of the Ni) was previously identified in an aqueous extract of *S. acuminata* using SEC monitorized on-line with ICP-MS, followed by off-line ESI-MS/MS analyses of the Ni-containing SEC-fractions (Schaumlöffel et al., 2003). In leaf extracts of several New Caledonia Ni hyper-accumulator plant species, Ni-Cit complexes were also found as the most prominent Ni containing ions detected by SEC-ESI-MS/MS (Callahan et al., 2012). In another Ni hyper-accumulator, *A. murale*, EXFAS studies indicated that Ni was found coordinated with Mal, His and other low molecular weight compounds in the plant sap and vasculature (McNear et al., 2010), and an *in silico* speciation study of the xylem sap of the hyper-accumulator *A. serpyllifolium* predicted approximately 18% of Ni bound to organic acids (Alves et al., 2011). However, a detailed study of the metal and ligand concentration in the xylem sap of Alyssum species treated with Ni for long periods indicated that most of the Ni in xylem sap of this species is present as the hydrated cation, and that the increases in His and other chelators may constitute only a short term response (Centofanti et al., 2013). The Ni(II)-NA complex is quite stable (log *K* = 16.1) and accordingly Ni has also been found complexed by NA in hyper-accumulators as well as model plant species. Nickel(II)-NA was found in the xylem sap (Mari et al., 2006) and plant extracts (Vacchina et al., 2003; Ouerdane et al., 2006) of *T. caerulescens*, in a water extract of the latex in *S. acuminata* (Schaumlöffel et al., 2003), and in leaf extracts of New Caledonia Ni hyper-accumulator plant species (Callahan et al., 2012), using SEC-ESI-MS/MS or SEC in combination with ICP-MS detection and off-line ESI-MS/MS. In Arabidopsis xylem sap, the Ni(II)-NA complex was also detected using both HPLC-MS (Xuan et al., 2006) and CE coupled to UV-VIS (Xuan et al., 2007). On the other hand, studies on natural variation among *Arabidopsis* accessions indicated that a Ni(II)-Mal complex may also be involved in translocation of Ni from roots to shoots (Agraval et al., 2013).

In the case of toxic metals such as Cd, complexation with organic ligands in xylem vessels may not be necessary, because toxicity exerted in this apoplastic, extracellular system is low and may not require a metal detoxification strategy. In fact, using ^113^Cd NMR analysis combined with a stable isotope (^113^Cd) labeling technique, Cd was found as an ionic form in *A. halleri* xylem sap (Ueno et al., 2008). However, several EXAFS spectroscopy studies indicate that Cd is coordinated with O or N ligands in *B. juncea* xylem sap (Salt et al., 1995) and with O in aerial parts of *T. caerulescens* (Küpper et al., 2004), *A. halleri* (Huguet et al., 2012) and *T. praecox* (Vogel-Mikus et al., 2010). In roots of *B. juncea*, a possible coordination of Cd with S ligands was also reported (Salt et al., 1995), with the bond length being similar to that of a purified Cd(II)-PC complex, supporting the occurrence of Cd(II)-PC complexes in plants (Salt et al., 1995). The occurrence of Cd association with PCs in the xylem sap of *B. juncea* has been proposed using SEC and off-line metal GFAAS, and using the retention times of several Cu-complexes with low molecular weight ligands (including PCs, GSH, Cys, organic acids, and inorganic anions) as a mean for identification (Wei et al., 2007b).

The chemical form of Al in xylem sap has been identified as the Al-Cit complex using ^27^Al-NMR analysis in several Al hyper-accumulators, including *Fagopyrum esculentum* (Ma and Hiradate, 2000), *Melastoma malabathricum* (Watanabe and Osaki, 2001) and *Camellia sinensis* (Morita et al., 2004). Since Al in roots was as an Al-oxalate (1:3) complex, ligand exchange from oxalate to Cit should occur in these plant species (Ma and Hiradate, 2000; Watanabe and Osaki, 2001).

## Metal species in phloem sap

The distribution of micronutrients to developing organs of plants depends to a great extent on phloem transport. Unlike xylem, phloem consists of columns of living cells. Metals are sparingly soluble at the alkaline pH values typical of the phloem sap (pH range from 7 to 8), and they are also highly reactive species, with some of them such as Fe undergoing easily changes of valence that favor the production of highly reactive oxygen species *via* Fenton reactions. Therefore, metal complexation with appropriate ligands can provide solubility and shielding during phloem transport of metals to the nutrient sinks.

In the phloem sap of *O. sativa*, Fe has been found predominantly (77%) associated with high molecular weight molecules (using a 3 kDa membrane filter; Nishiyama et al., 2012). In this study, Fe-containing compounds or complexes of 10–30 kDa and the Fe(III)-DMA complex were detected in the phloem sap using anion exchange HPLC separation followed by an identification based on Fe determination and the comparison of the retention time with those of standards, in combination with exact molecular mass and Fe isotopic signature obtained using ESI-MS(TOF). A protein capable to bind Fe was described in the phloem sap of *R. communis* (ITP; Iron Transport Protein; Krüger et al., 2002) using two-dimensional gel electrophoresis protein separation (2-DE SDS-PAGE) followed by electro-blotting to PVDF membranes and staining of Fe-containing proteins with Ferene. Recently, two more low molecular weight Fe-binding proteins were also identified in *L. texensis* phloem sap using a similar approach combined with Fe affinity chromatography, although none of them are considered candidates for Fe transport (Lattanzio et al., 2013). The Fe(II)-NA complex has not been found so far in the phloem sap, although *in silico* and/or *in vitro* studies support that the Fe-NA complex is likely to occur at the neutral to basic pH values of the phloem sap (von Wirén et al., 1999; Rellán-Álvarez et al., 2008), and YSL transporters able to transport Fe-NA complexes have been described in *Arabidopsis* and *O. sativa* phloem vascular tissues (Curie et al., 2009). Perhaps NA is only important in Fe phloem loading (Schuler et al., 2012), and once in that compartment Fe may be transported in another form such as bound to proteins.

Almost all Zn in the *O. sativa* phloem sap was found associated with low molecular weight molecules when a 3 kDa membrane filter was used as a cut-off (Nishiyama et al., 2012). In this study, Zn was identified as the Zn(II)-NA complex, using SEC and off-line Zn determination and off-line ESI-MS, based on exact molecular mass, isotopic signature, and retention time. In *L. texensis* phloem sap, four low molecular weight Zn-binding proteins were identified using 2-DE SDS-PAGE, nanoLC-MS/MS and Zn affinity chromatography, one of them being a metallothionein-like protein type 2B, but they were not considered as good candidates for Zn transport (Lattanzio et al., 2013).

Regarding Cu, the phloem sap of *O. sativa* has been shown to contain the complexes Cu(II)-NA, Cu(II)-His and high-molecular-weight compounds >3 kDa (the latter being at least 30% of the total Cu) (Ando et al., 2013). Copper(II)-NA and Cu(II)-His were identified using SEC combined with both off-line Cu determination by GFAAS and molecular detection of the complex in the major Cu-containing fractions. Copper-containing proteins detected in phloem sap so far include Cu/Zn-superoxide dismutase, a Cu-chaperone (CCH homolog) and several MTs in *O. sativa* (Aki et al., 2008) and *L. texensis* (Lattanzio et al., 2013).

A Cd-containing complex has not been directly identified in the phloem sap so far. However, 90% of the Cd in the phloem sap from Cd-treated *O. sativa* plants was found in a complexed form using SEC-ICP-MS (Kato et al., 2010). Based on the elution times of *in vitro* prepared Cd-complexes with glutathione and several PCs and on the changes caused on Cd elution by sap digestion with proteinase K, it was proposed that Cd was found associated with a 13 kDa protein and SH-containing compounds. Cadmium has also been associated with S in the phloem and companion cells of *A. thaliana* using energy-dispersive X-ray microanalysis (van Belleghem et al., 2007). This, along with the occurrence of PCs in phloem of Cd-treated plants (see above) suggest the occurrence of Cd-PC complexes in the phloem sap.

Much less information is available on the chemical forms of other metals in the phloem sap. In *R. communis*, Mn was detected in association with low molecular peptides (van Goor and Wiersma, 1976), while Ni was shown to be complexed with negatively charged organic compounds with a molecular weight in the range of 1000–5000 Da (Wiersma and van Goor, 1979).

## Metal species in other fluids

The ESL is a fluid that facilitates metal transport from the seed coat to the embryo. Iron speciation in isolated *P. sativum* ESL was achieved using an integrated analytical approach, combining XANES, HILIC-ICP-MS, and HILIC-ESI-MS (Grillet et al., 2014). The application of the XANES technique indicated that most of the Fe was present as Fe(III), probably associated to carboxylates—although the XANES spectra of Fe-Cit and Fe-Mal could not be distinguished—with only a minor amount of Fe(II) species being present. Most (88%) of the Fe occurring in the ESL was found as the species Fe(III)_3_Cit_2_Mal_2_, Fe(III)_3_Cit_3_Mal (both are mixed ligand species) and Fe(III)Cit_2_; only a minor amount of Fe(II)-NA was found using HILIC separation coupled to the two cited MS detectors. Metal species were identified based on elution time, Fe determination, exact mass determination, isotopic signature, and MS^2^ fragmentation pattern of the Fe species as identification tools. Pea embryos are capable of reducing Fe(III) in these complexes by effluxing to the ESL high amounts of ascorbate that chemically reduce Fe(III) from the Fe-Cit and Fe-Mal complexes. Ascorbate efflux also occurs in *A. thaliana* embryos and is significantly decreased, along with Fe concentrations, in ascorbate deficient mutants (Grillet et al., 2014). These data provide support for a new local Fe transport system that may play a major role to control Fe loading in seeds in dicotyledonous plants.

Apoplastic fluid composition is determined by the balance of import via xylem, absorption by cells, and export by phloem, and plays important roles in the transport and storage of mineral nutrients (Sattelmacher, 2001). However, as far as we know, there are no reports tackling direct metal speciation on apoplastic fluids. *In silico* calculations have been carried out to speciate Fe in leaf apoplastic fluid of *B. vulgaris* (López-Millán et al., 2000; Larbi et al., 2010) and field-grown *P. communis* (López-Millán et al., 2001) using experimental concentrations of Fe, inorganic ions, carboxylates, sugars and amino acids measured from fluids isolated from Fe-sufficient and Fe-deficient plants. In both plant species, Fe was predicted to occur in the leaf apoplastic fluid as Fe-Cit complexes, with FeCitOH and Fe(III)Cit_2_ being the major species. The effect of Fe deficiency altered the balance between these two Fe-Cit species and the contribution of Fe(III)Cit_2_ increased with Fe deficiency in *B. vulgaris*, whereas in *P. communis* Fe(III)Cit_2_ was lower in Fe-deficient trees.

## Summary and concluding remarks

**Figure 1:**
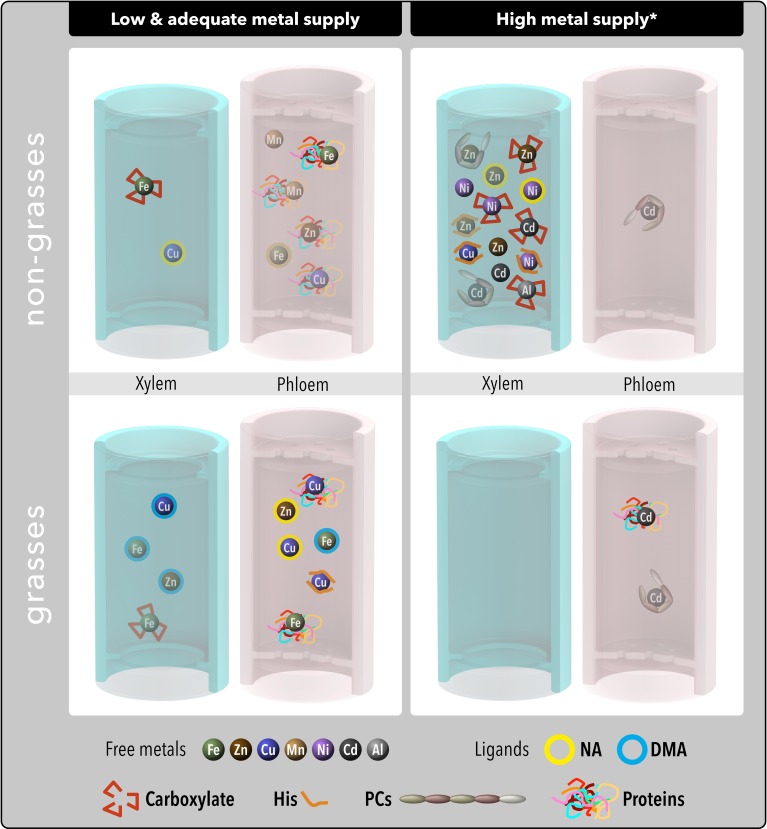
**Schematic representation showing the metal species found in the xylem and phloem saps of non-grass (upper panels) and grass plant species (lower panels) grown at low and adequate metal supply (left panels) or at high metal supply (right panels)**. Metals occurred in free ionic forms and in complexes with different ligands such as nicotianamine (NA), 2′-deoxymugineic acid (DMA), carboxylates (e.g., citrate), histidine (His), phytochelatins (PCs) and proteins. Putative metal species proposed to occur in plant fluids as supported by strong evidence from indirect approaches (e.g., molecular biology and others) are shown with faded symbols. ^*^Most of the data with high metal supply have been described in metal hyper-accumulator plant species.

The new developments in MS techniques and the increased use of X-ray spectroscopy methods at synchrotron facilities have permitted the discovery of a number of natural metal species (*ca*. 19) in xylem and phloem saps (Figure 1; clear symbols). Moreover, evidence supporting the occurrence in such fluids of further putative metal species (*ca*. 15) has been provided by means of physiological studies, many of them applying molecular biology tools (Figure 1; faded symbols). Approximately 50% of the confirmed metal species and 27% of the putative ones have been reported in the xylem sap of plants (mostly hyper-accumulators) treated with high metal supply including Zn, Ni, and Cd. The second most abundant group of metal species has been described in the phloem sap of plants (including grasses) grown at low and adequate metal supply, and accounts for 37% of the confirmed metal species and 33% of those considered putative. This second (phloem) group includes mainly species containing Fe and Cu. Metal carboxylate complexes have always been found in xylem, whereas metals associated with proteins or high molecular weight compounds have been reported in phloem. Complexes with high affinity metal ligands (e.g., NA for Cu and Zn, PSs for Fe and PCs for Cd) have been commonly found in phloem sap, where complexation is needed to ensure metal solubility at the existing high pH as well as to avoid uncontrolled binding of metals in living cells, ensuring metal transport and delivery to sinks. Also, high affinity metal ligands have been found as metal carriers in the xylem sap when plants are grown with low or high metal supply. When metal availability is scarce, organic ligand-assisted xylem transport (e.g., Fe complexed by carboxylates or DMA) can increase transport efficiency, because complexation ensures metal delivery, while avoiding unwanted reactions and scavenging any metal pools occurring in the apoplast. Under conditions of high metal supply, organic ligand-assisted xylem transport (e.g., Ni-NA or Cd-PCs) may attenuate the toxicity derived of the exceptionally high metal concentrations as well as to ensure a correct delivery of other metals.

Most of the knowledge on metal complexes in xylem and phloem has been gained using plants (mostly hyper-accumulators) grown on hydroponics with either a short-time excess metal supply treatment or with a established metal deficiency followed by a short-time metal re-supply treatment (the latter in the case of Fe or Zn). Since these conditions are far from the natural ones, some of the reported metal species can differ from the actual complexes existing in plants in natural conditions (Centofanti et al., 2013). The reason to use such growth conditions is to force upwards the metal concentrations usually found in plant fluid samples (which rarely are >50 μM; Table 1). These low concentrations make particularly challenging the speciation of metals in plant fluids in natural conditions. Also, plant fluids contain ions, sugars, proteins and other biomolecules along with the metal of interest and the rest of metals. This leads to a delicate balance among several metal species, which can include free metal ions and metal complexes having a great diversity of size, charge, and stability. Therefore, the direct analysis of the metal species in plant fluids requires of highly conservative (avoiding any alteration of sample that can damage metal species), sensitive and selective analytical techniques. Unfortunately, none of different techniques available comply with all of these characteristics.

X-ray spectroscopy methods are non-invasive and apparently conservative, although damage of the sample can occur, but they are much less sensitive than ICP-MS and much less selective than ESI-MS/MS. In contrast, MS-based techniques need to be combined with separation methodologies that are inherently invasive since they carry out the separation of the sample in fractions of different characteristics (e.g., molecular size in SEC), and this can significantly affect the distribution of metal chemical forms, particularly in the case of weak metal complexes specially sensitive to changes in pH and ligand-to-metal ratios. For these reasons, metal species found using MS techniques usually require confirmation using alternative separation methods to validate the actual existence of such metal-complexes in the samples, and sometimes alternative detection methods (e.g., NMR of isolated fractions) are needed to elucidate identity. Ultimately, the occurrence of the metal complexes identified *via* MS should be also confirmed in intact samples with XAS methods. On the other hand, the use of X-ray spectroscopy methods requires having pure standards of putative metal species, because data interpretation relies on linear combination fitting procedures where the sample spectra is fitted using those of standard compounds. Also, XAS techniques can hardly give any information on well-defined metal species when the sample contains several species with similar XANES spectra (e.g., metal complexes with carboxylates). Therefore, XAS also needs complementary techniques (e.g., ESI-MS/MS) to provide putative metal species as well as to confirm the correct identity of the metal species occurring in the samples.

The path of any given metal to its final destination (e.g., the chloroplast) involves transport across multiple membranes mediated by different transporter proteins and most likely metal complexation by different ligands in each compartment within the path. Although a complete picture of this complex process is still lacking even for one metal, an increasingly more complete and accurate knowledge of the metal species in plant fluids would be achieved performing studies that integrate molecular biology approaches, untargeted analyses of plant fluids using complementary MS-based and NMR techniques, and targeted XAS methods. The limits of detection and quantification of the techniques actually used are still far from ideal to analyze fluids coming from plants grown in natural conditions, and therefore more analytical efforts are required to completely decipher the metal species transported in plant fluids.

### Conflict of interest statement

The authors declare that the research was conducted in the absence of any commercial or financial relationships that could be construed as a potential conflict of interest.
